# Pneumothorax induced by cocaine inhalation revealing bronchiectasis with bullous emphysema

**DOI:** 10.1016/j.radcr.2025.11.009

**Published:** 2025-12-04

**Authors:** Marwane El Arabi, Wael EL Fergui, Anass Litim, Salma Abddaimi, Safaa Kachmar, Younes Oujidi, Houssam Bkiyar

**Affiliations:** a Department of ICU, University Hospital Mohammed 6, Oujda, Morocco; b Department of Radiology, University Hospital Mohammed 6, Oujda, Morocco

**Keywords:** Cocaine inhalation, Spontaneous pneumothorax, Bronchiectasis, Bullous emphysema, Pulmonary tuberculosis

## Abstract

Cocaine inhalation is a known cause of various pulmonary complications, both acute and chronic, including spontaneous pneumothorax, which may be exacerbated by unrecognized underlying lung diseases such as bronchiectasis or emphysema. We report the case of a 33-year-old man with a history of chronic cocaine use who presented to the emergency department with dyspnea and right-sided chest pain. Imaging revealed a right-sided tension pneumothorax associated with bronchiectatic changes and bullous emphysema, likely secondary to previous pulmonary tuberculosis. The patient was treated conservatively with chest drainage, resulting in marked clinical improvement. This case underscores that cocaine-induced pneumothorax, though rare, should be suspected in young adults presenting with acute respiratory distress in the absence of trauma. Deep inhalation, Valsalva maneuvers, and barotrauma may contribute to alveolar rupture, while cocaine use can unmask or worsen chronic pulmonary damage. Clinicians should systematically assess for underlying structural lung disease, especially post-tuberculosis changes, to guide management and prevent recurrence through pleurodesis or drug cessation.

## Introduction

Cocaine is an illicit stimulant whose inhalation can cause multiple acute and chronic pulmonary complications due to its vasoactive, inflammatory, and barotrauma-inducing effects [1]. Reported respiratory manifestations include alveolar hemorrhage, acute lung injury, pneumonitis, and spontaneous pneumothorax [1]. The risk is heightened by the inhalation technique, which often involves deep inspiration and prolonged breath-holding, increasing intrathoracic pressure. In chronic users, cocaine inhalation may also contribute to the development or exacerbation of structural lung disease, including emphysema and bronchiectasis. Bullous emphysema, in particular, can predispose to recurrent pneumothorax [2]. We report the case of a patient who developed a spontaneous pneumothorax following cocaine inhalation, which subsequently led to the incidental discovery of underlying bronchiectasis with multiple emphysematous bullae, most likely related to postprimary pulmonary tuberculosis.

## Presentation of the case

A 33-year-old man presented to the emergency department with shortness of breath and right-sided chest pain. He denied neck pain, dysphagia, odynophagia, or dysphonia. He reported having inhaled a larger-than-usual dose of cocaine a few nights prior to presentation. There was no history of trauma or vigorous exertion. He complained of mild rhinorrhea but denied cough or vomiting. He was not on any regular medication but reported frequent use of illicit drugs, including cocaine and ecstasy.

On examination, the patient was alert but in respiratory distress, with difficulty responding. Vital signs were as follows: heart rate 130 bpm, blood pressure 125/55 mmHg, respiratory rate 25 breaths/min, oxygen saturation 80% on room air, and afebrile. No subcutaneous crepitus was palpable in the neck or chest. The remainder of the physical examination was unremarkable.

Arterial blood gases revealed severe hypoxemia (PaO₂ 55 mmHg) and hypercapnia (PaCO₂ 57 mmHg). Renal function was normal, and blood glucose was 111 mg/dL. Complete blood count showed leukocytosis (WBC 23,450/µL), hemoglobin 12.1 g/dL, and platelets 509,000/µL. Electrocardiogram demonstrated normal sinus rhythm without arrhythmia or ischemic changes.

**Fig. 1:**
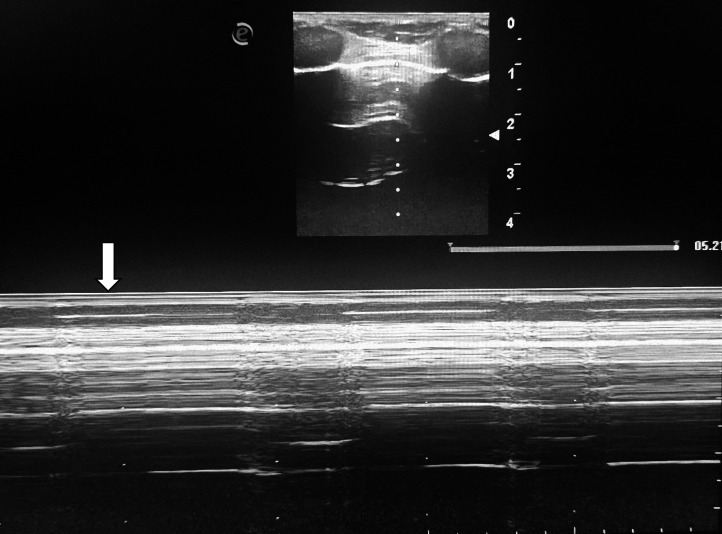
M-mode lung ultrasound showing the barcode sign (absence of pleural sliding), consistent with pneumothorax (with arrow).

**Fig. 2:**
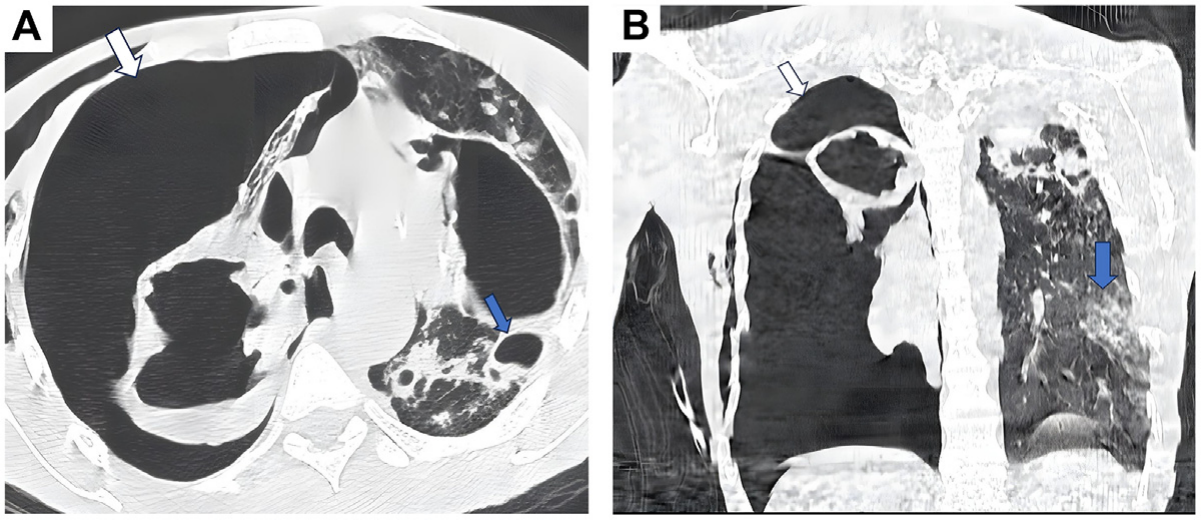
A. Axial chest CT scan (lung window) showing a large right-sided tension pneumothorax (white arrow) with collapse of the right lung. Note the presence of cystic and bronchiectatic changes with apical pseudo-cavitary lesions and areas of bullous emphysema, suggestive of chronic postinfectious sequelae (blue arrow). B. Coronal chest CT scan (lung window) showing a massive right-sided tension pneumothorax (white arrow) with near-total collapse of the right lung and leftward mediastinal shift. The left lung demonstrates fibro-bronchiectatic changes and parenchymal opacities consistent with postinfectious sequelae, likely secondary to prior pulmonary tuberculosis (Blue arrow).

Bedside chest ultrasound demonstrated the barcode sign with absence of pleural sliding, consistent with pneumothorax (Fig. 1). Chest CT confirmed a large right-sided tension pneumothorax, along with cystic and moniliform bronchiectatic foci and bilateral apical pseudo-cavitary lesions with signs of superinfection. There were also findings of infectious respiratory bronchiolitis with a predominant branching nodular pattern. Overall, the radiologic features were highly suggestive of postprimary pulmonary tuberculosis with acute superinfection. Additional pulmonary sequelae of cocaine use, such as granulomas, emphysema, and bullae, were also evident (Figs. 2A and B).

**Fig. 3:**
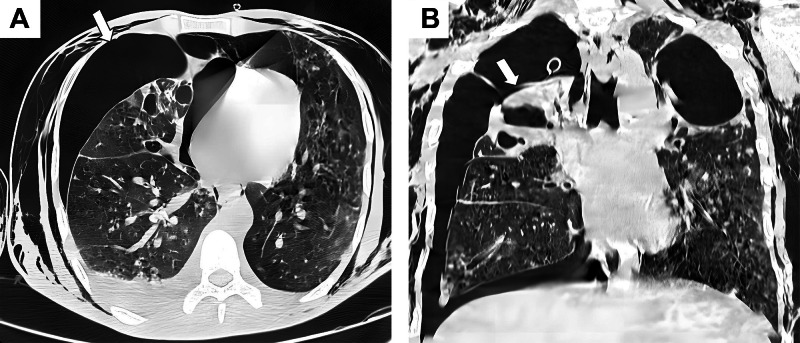
(A) Axial chest CT scan (lung window) obtained after chest drainage, demonstrating marked regression of the right pneumothorax (white arrow) and partial re-expansion of the right lung. (B) Coronal chest CT scan (lung window) obtained after chest drainage showing marked regression of the right pneumothorax (white arrow) and partial re-expansion of the right lung. The chest tube is seen in place. Note the presence of residual bullous and bronchiectatic changes in the right lung parenchyma and fibrotic alterations in the left lung, consistent with chronic postinfectious sequelae.

The cardiothoracic surgery team determined that the patient did not meet criteria for immediate surgical intervention. He was transferred to the intensive care unit, where he was managed conservatively with chest drainage and monitored for 48 hours. During this period, his respiratory function improved, though the pneumothorax did not fully resolve. A follow-up CT demonstrated marked regression of the pneumothorax (Figs. 3A and B).

The patient made a significant clinical recovery and was discharged on room air after 4 days of hospitalization. He was prescribed a 4-week course of amoxicillin/clavulanate (1000 mg) for possible superinfection associated with cavitary lesions. Follow-up was arranged with pulmonology for interval imaging and possible pleurodesis, given the high recurrence risk. Tuberculosis therapy was initiated after diagnostic confirmation. The patient declined pleurodesis during this admission, opting for lifestyle modification, including cessation of cocaine use, to reduce recurrence risk.

## Discussion

Over the past 2 decades, recreational cocaine abuse has increased significantly, accompanied by the emergence of new routes of administration [3]. Crack cocaine, the freebase and heat-stable form of the drug, is most commonly smoked using glass or metal pipes or mixed with tobacco or marijuana [4]. The euphoric effects of smoked cocaine occur rapidly, typically within seconds [4]. Its low cost and ease of use have contributed to its growing popularity, particularly among young adults [5].

The lungs are the primary organs exposed to the combustion products of crack cocaine, and thermal injury to the airways can be severe [4]. Following inhalation, drug particles reach the alveoli and deposit throughout the pulmonary tissue, leading to alveolar septal thickening and destruction [6]. A further predisposing factor for bullous disease is the alveolar deposition of talc particles commonly found in cocaine, which triggers a foreign-body granulomatous reaction and results in air trapping and bullae formation.

Acute pulmonary complications of cocaine inhalation include cough, hemoptysis, pneumothorax, pneumomediastinum, pneumopericardium, and hemothorax. Users often inhale deeply and perform a Valsalva maneuver to enhance the drug’s psychoactive effects. This practice, combined with barotrauma induced by vigorous coughing, causes a marked rise in intra-alveolar pressure that can lead to alveolar rupture and dissection of air into peribronchiolar connective tissue, potentially culminating in pneumothorax [7].

Our patient developed a large right-sided tension pneumothorax following intranasal cocaine use, without trauma or other precipitating factors. The presence of cystic and moniliform bronchiectatic foci with apical pseudo-cavitary lesions raised the possibility of underlying structural lung disease. The radiologic features were highly suggestive of postprimary pulmonary tuberculosis complicated by superinfection, which likely predisposed the patient to alveolar rupture. Cocaine-induced pulmonary damage, including emphysema, bullous disease, and talc-related granulomas, may have contributed to lung parenchymal fragility.

Several studies suggest an association between cocaine inhalation and pulmonary tuberculosis. Cocaine use can induce direct respiratory epithelial injury, impair mucociliary clearance, and promote local inflammation, facilitating *Mycobacterium tuberculosis* infection or reactivation of latent disease [8]. Additionally, habitual cocaine users often have immunosuppression due to poor nutrition, HIV coinfection, or polysubstance abuse, increasing susceptibility to tuberculosis [9]. The radiologic findings in our patient, characteristic of postprimary tuberculosis, may have been exacerbated by chronic cocaine-related injury. This overlap complicates diagnosis, as both entities can present with cavitation, granulomatous inflammation, and secondary infection. Cocaine inhalation should thus be recognized as a risk factor for structural lung damage and pneumothorax, as well as a potential cofactor in tuberculosis reactivation.

Management of cocaine-induced pneumothorax generally follows standard guidelines, with supplemental oxygen, observation, or chest tube drainage depending on severity and stability [10]. In our patient, chest drainage resulted in clinical improvement with significant pneumothorax regression on follow-up imaging. However, recurrence remains a major concern in patients with structural lung disease or continued drug use. Pleurodesis is a recognized preventive measure but was declined by our patient in favor of lifestyle modification.

This case highlights the importance of considering illicit drug use in the differential diagnosis of spontaneous pneumothorax, especially in young adults without trauma. It also underscores the potential interplay between recreational drug abuse and chronic infectious diseases, such as tuberculosis, in exacerbating pulmonary complications.

## Conclusion

This case illustrates the complex interplay between illicit drug use and chronic pulmonary disease. Cocaine inhalation can precipitate acute complications such as spontaneous pneumothorax while contributing to or revealing underlying structural lung damage, including bronchiectasis and bullous emphysema. The coexistence of postprimary pulmonary tuberculosis further complicates diagnosis and management, emphasizing the need for comprehensive evaluation of patients presenting with unexplained respiratory symptoms and a history of drug use. Management should follow standard pneumothorax guidelines while addressing comorbidities and considering preventive strategies, such as pleurodesis, in high-risk cases. This case emphasizes the importance of integrating clinical suspicion, imaging findings, and substance use history to optimize care and reduce recurrence risk.

## Patient consent

Written informed consent was obtained from the patient for publication and any accompanying images. A copy of the written consent is available for review by the Editor-in-Chief of this journal on request.
